# Molecular genetic diversity and population structure analyses of rutabaga accessions from Nordic countries as revealed by single nucleotide polymorphism markers

**DOI:** 10.1186/s12864-021-07762-4

**Published:** 2021-06-12

**Authors:** Zhiyu Yu, Rudolph Fredua-Agyeman, Sheau-Fang Hwang, Stephen E. Strelkov

**Affiliations:** Department of Agricultural, Food and Nutritional Science, University of Alberta, Edmonton, AB T6G 2P5 Canada

**Keywords:** *Brassica*, SNP, AMOVA, Population differentiation, PCoA, UPGMA and NJ

## Abstract

**Background:**

Rutabaga or swede (*Brassica napus ssp. napobrassica* (L.) Hanelt) varies in root and leaf shape and colour, flesh colour, foliage growth habits, maturity date, seed quality parameters, disease resistance and other traits. Despite these morphological differences, no in-depth molecular analyses of genetic diversity have been conducted in this crop. Understanding this diversity is important for conservation and broadening the use of this resource.

**Results:**

This study investigated the genetic diversity within and among 124 rutabaga accessions from five Nordic countries (Norway, Sweden, Finland, Denmark and Iceland) using a 15 K single nucleotide polymorphism (SNP) *Brassica* array. After excluding markers that did not amplify genomic DNA, monomorphic and low coverage site markers, the accessions were analyzedwith 6861 SNP markers. Allelic frequency statistics, including polymorphism information content (PIC), minor allele frequency (MAF) and mean expected heterozygosity ($\overline{H}$e) and population differentiation statistics such as Wright’s F-statistics (*F*_*ST*_) and analysis of molecular variance (AMOVA) indicated that the rutabaga accessions from Norway, Sweden, Finland and Denmark were not genetically different from each other. In contrast, accessions from these countries were significantly different from the accessions from Iceland (*P* < 0.05). Bayesian analysis with the software *STRUCTURE* placed 66.9% of the rutabaga accessions into three to four clusters, while the remaining 33.1% constituted admixtures. Three multivariate analyses: principal coordinate analysis (PCoA), the unweighted pair group method with arithmetic mean (UPGMA) and neighbour-joining (NJ) clustering methods grouped the 124 accessions into four to six subgroups.

**Conclusion:**

Overall, the correlation of the accessions with their geographic origin was very low, except for the accessions from Iceland. Thus, Icelandic rutabaga accessions can offer valuable germplasm for crop improvement.

**Supplementary Information:**

The online version contains supplementary material available at 10.1186/s12864-021-07762-4.

## Background

*Brassica napus ssp. napobrassica* (L.) Hanelt*,* called ‘rutabagge’ in Sweden, ‘rutabaga’ in the USA and Canada, and ‘swede’ in the UK, New Zealand and Australia, is a cool-weather root crop thought to have been derived from the natural or spontaneous hybridization between *B. rapa* (turnip) and *B. oleracea* (cabbage or kale) [1]. Rutabaga is often assumed to have originated in Sweden, but may have come from Finland [2, 3]. Nevertheless, it was distributed from Sweden (where it grew in the wild before 1400) to England, Germany and other European countries around the end of the eighteenth century [4] and was introduced to North America by European immigrants in the early nineteenth century [5]. Therefore, the Nordic countries are considered the center of rutabaga domestication and diversity.

Rutabagas are grown for use as a table vegetable and as fodder for animals [3]. The roots are rich in vitamins A, C and fibre; are low in calories and have trace amounts of vitamin B_1_, B_2_, potassium, calcium, magnesium and iron [3, 6]. Like most cruciferous vegetables, they have antioxidant and anti-cancer properties [7]. The leaves have much higher levels of protein (17–18%) than the roots (0.6–2.0%) [8, 9]. However, most of the components are non-protein nitrogen (urea and ammonia), which can be converted into protein by microbes in the stomach of ruminants, but not in pigs [10]. Rutabagas vary considerably in morphology, disease resistance, seed yield and quality parameters such as erucic acid and glucosinolate content [3, 11]. Breeding efforts have targeted root appearance and flesh colour, earliness, drought tolerance, improvement in resistance to diseases, broadening genetic diversity and quality traits associated with the seeds [3, 6, 12–15]. Quantitative traits such as root length, diameter and fresh weight are also of interest for crop improvement [16].

Genetic variation in plants is a key pillar of biodiversity and provides the resources for the development of new and improved cultivars with desirable characteristics [17]. In addition, studying diversity in natural plant populations makes it possible to understand genetic exchange or gene flow within and between populations [18]. Many genetic diversity studies have utilized simple sequence repeat (SSR) and single nucleotide polymorphism (SNP) markers due to their abundance and co-dominant nature. However, PCR amplification of genomic DNA using SSR markers can produce sequence artifacts because of errors in Taq DNA polymerase activity and the formation of chimeric and heteroduplex molecules [19–21]. The production of artifacts, particularly in the case of highly polymorphic SSR markers, can cause difficulties in allele size calling [22]. Alleles of the same sized products may have different sequences [23]. This can also affect the quality of genotyping data. Random amplified polymorphic DNA (RAPD) markers are dominant markers with low reproducibility and accuracy, while random fragment length polymorphism (RFLP) markers have a low discrimination power and can be costly [24].

In contrast, SNPs arise because of point mutations and hence most SNPs are biallelic, which leads to greater accuracy in genotyping; these markers also offer the advantage of co-dominance. In addition, SNP-based systems lend themselves to automation, and hence a larger number of markers (tens of thousands or higher) can be screened within a shorter time in comparison with the use of SSR markers [25]. The high heritability of SNPs makes them the marker of choice for studying genetic diversity and phylogeny in crop species with ancient genome duplications such as *B. napus* [26]. A major drawback is that SNP calling is difficult for polyploid species such as *B. napus* [25]. In addition, SNP markers used for genetic diversity studies should be neutral or be present in non-coding regions to eliminate bias introduced by selection when inferring population structure. Therefore, SNP arrays used for genotyping require extensive validation to confirm their usefulness for general application. Genome resequencing is an alternative to array-based methods and generally yields over a million SNP markers [27–30].

Previous molecular studies indicated that spring oilseed rape, winter oilseed rape, fodder and vegetable types, and rutabagas formed separate clusters of *B. napus* [31–33]. Bus et al. [31] used 89 SSR markers to estimate genetic diversity in 509 *B. napus* inbred lines, of which 73 were swedes or rutabagas. Similarly, Diers and Osborn [32] used 43 RFLP markers to group 83 *B. napus* lines including two rutabagas. Mailer et al. [33] reported that a set of 100 RAPD markers could identify four rutabaga accessions among 23 cultivars of *B. napus*. Zhou et al. [27] used 30,877 SNP markers to differentiate 300 *Brassica* accessions into spring, semi-winter and winter ecotypes. Gazave et al. [28] genotyped 782 *B. napus* accessions with 30,881 high quality SNP markers and reported three major subpopulations, of which the highest variance was found in the spring and winter samples. Whole genome sequencing has indicated that winter oilseeds, which include rutabagas, may be the original form of *B. napus* and that this crop may have multiple origins [29, 30].

One hundred seventy-one rutabaga accessions are available (assessed on January 11th, 2021) from the Nordic Genetic Resource Center, Alnarp, Sweden. Of these, 145 accessions are from the Nordic countries, 20 are from France, four are from Germany and one accession each is from Estonia and the United Kingdom. Many of these are landraces with great genetic variability that can be exploited in rutabaga and other *Brassica* breeding programs around the world. The genetic diversity and variability that exist within and among rutabaga accessions and populations from the Nordic countries have not been examined. Understanding this diversity is important for conservation and broadening the use of this important resource. Therefore, the aim of the present study was to use high-throughput genotyping with *Brassica* SNP markers to estimate genetic diversity in rutabaga accessions from five Nordic countries (Norway, Sweden, Finland, Denmark and Iceland).

## Results

### SNP marker characteristics

Thirteen thousand seven hundred four SNP markers on the 15 K SNP *Brassica* chip were used to screen the 124 rutabaga accessions and three rutabaga cultivars. Among these, 31% (4213 SNPs) were monomorphic, 5% (701 SNPs) were low coverage site markers, and 14% (1929 SNPs) were missing data points for > 5% of the accessions. Thus, filtering removed ≈ 50% of the SNP markers, while the remaining ≈ 50% (6861 SNPs) were retained for the diversity analysis. This comprised 4390 A-genome and 2471 C-genome SNP markers.

### Allelic patterns and genetic diversity indices among and within populations

**Fig. 1:**
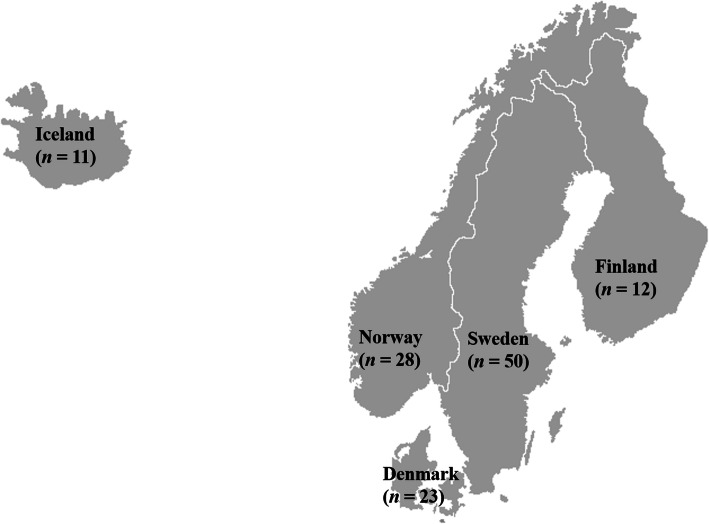
The origin and sample sizes per country of the 124 rutabaga accessions used in this genetic diversity study. The Nordic region (Norway, Sweden, Finland, Denmark and Iceland) is often cited as the center of domestication and diversity of rutabaga

**Fig. 2:**
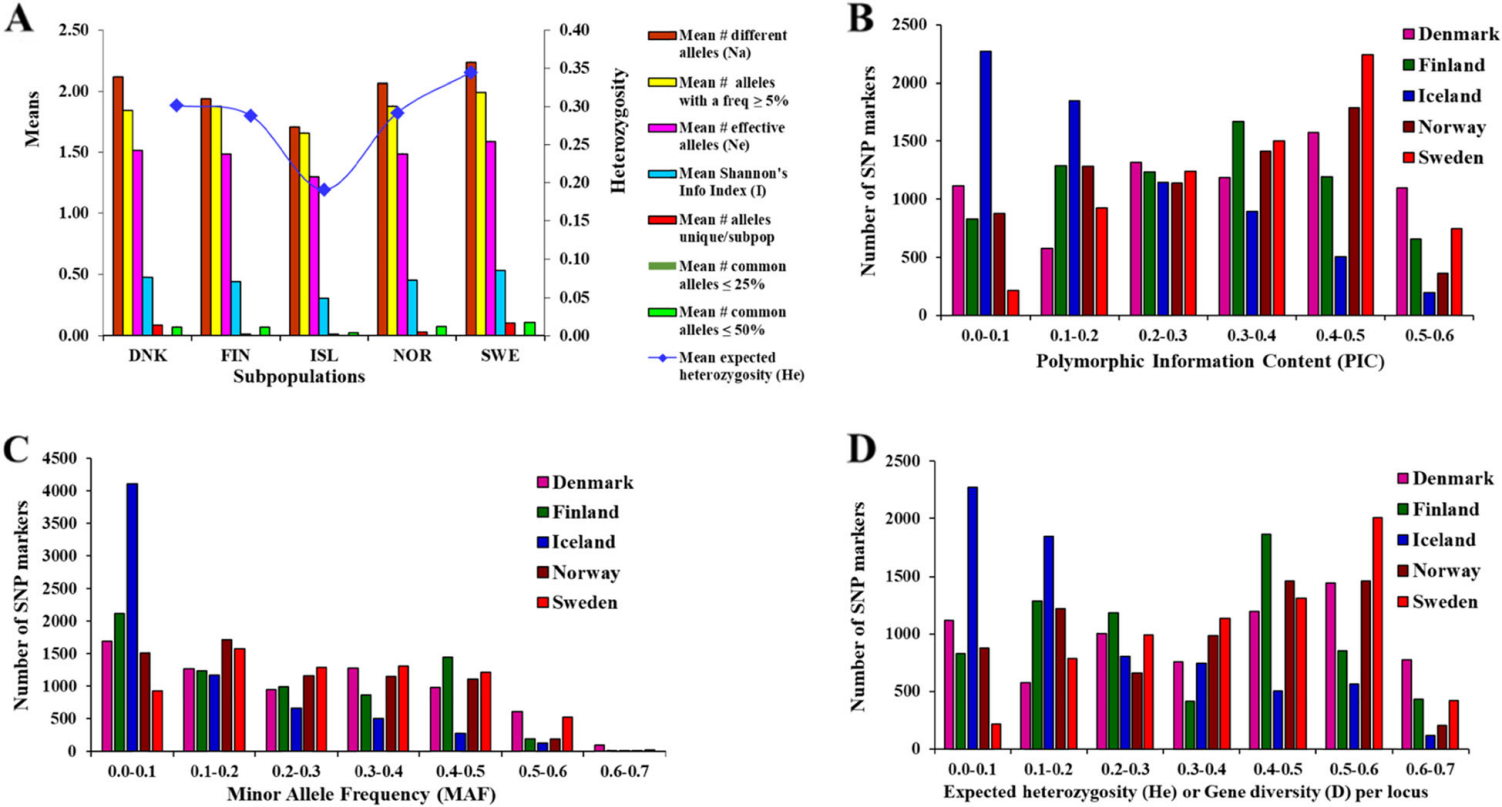
Distribution of allele frequency-based genetic diversity statistics **(A)**, Polymorphic Information Content (PIC) **(B)**, Minor Allele Frequency (MAF) **(C)**, and Expected heterozygosity (*He*) or gene diversity **(D)** of 6861 SNP markers across 124 rutabaga accessions from Norway, Sweden, Finland, Denmark and Iceland

Figure 1 shows the origin and sample sizes of the rutabaga accessions used for this study. Allelic patterns and genetic diversity summary statistics at any given locus or averaged across the 6861 SNP loci for the rutabaga accessions separately for each country and for the whole collection are presented in Table S1 and Fig. 2A to D.

The proportion of polymorphic loci (%*P*) detected separately for the NOR-, SWE-, FIN- and DNK- subpopulations was significantly higher (range 88.5–99.6%) than for the ISL-subpopulation (67.9%) (*P* < 0.05) (Table S1). The mean number of alleles per locus (*Na*) was highest in the SWE-subpopulation (2.236 ± 0.005) and lowest in the ISL-subpopulation (1.707 ± 0.006) (Table S1). Similarly, the mean number of effective alleles per locus (*Ne*) and Shannon’s information index (*I*) were significantly higher in the SWE-subpopulation (1.590 ± 0.004 and 0.535 ± 0.002, respectively) compared with the ISL-subpopulation (1.299 ± 0.004 and 0.305 ± 0.003, respectively) (Table S1). In addition, the mean number of alleles with a frequency ≥ 5% (*Na Freq* ≥ 5%) and mean number of common alleles found in ≤50% of the subpopulations (*Na common* ≤ 50%) were lowest for the ISL-subpopulation (Fig. 2A). Thus, most of the genetic diversity indices for the NOR-, SWE-, FIN- and DNK-subpopulations were not significantly different from each other. They were, however, all significantly different from the ISL-subpopulation (*P* < 0.05).

The diversity of the SNP markers expressed as the polymorphic information content (PIC) is presented in Fig. 2B. The number of markers with PIC > 0.2 was highest for the SWE-subpopulation (5725 ≈ 83%) and DNK-subpopulation (5170 ≈ 75%), intermediate for the FIN- and NOR-subpopulations (4701–4726 ≈ 69%), and lowest among for the ISL-subpopulation (2742 ≈ 40%). The PIC averaged across the 6861 SNPs separately for each population followed similar patterns as the allelic and genetic diversity, with the highest PIC occurring in the SWE-subpopulation (0.35) and the lowest in the ISL-subpopulation (0.18).

The number of SNP markers with minor allele frequency (MAF) ≤ 0.1 was of the order ISL- (4106 ≈ 60%) > FIN- (2115 ≈ 31%) > DNK- (1690 ≈ 25%) > NOR- (1518 ≈ 22%) > SWE-subpopulations (933 ≈ 14%). Thus, the frequency of minor alleles was highest for the ISL-subpopulation, intermediate for the FIN-, DEN- and NOR-subpopulations, and lowest for the SWE-subpopulation (Fig. 2C).

The expected heterozygosity per locus (*H*_*e*_), also called gene diversity (*D*), followed similar patterns as the rest of the parameters measured with the exception of the MAF (Fig. 2D). Analyses of the gene pool structure ($\overline{H}$_*e*_, expected heterozygosity averaged over all 6861 loci) of the rutabaga accessions from each country suggested that there was no significant difference in the genetic variability of the rutabaga accessions from Sweden (0.345 ± 0.002), Denmark (0.301 ± 0.002), Norway (0.292 ± 0.002), and Finland (0.288 ± 0.002). These accessions were, however, genetically different from the accessions from Iceland (0.191 ± 0.002) (Table S1).

### Genetic differentiation among regions, populations and within accessions

Pairwise comparisons of population differentiation using the fixation statistics index (*F*_*ST*_) are presented in Table 1. The *F*_*ST*_ values for all 10 pairwise combinations of all five subpopulations ranged from 0.032 to 0.133. Pairwise *F*_*ST*_ values for NOR/SWE, NOR/FIN and SWE/FIN ranged from 0.032 to 0.067 (lowest); the values for NOR/DNK, SWE/DNK and FIN/DNK ranged from 0.050 to 0.88 (intermediate); whereas the *F*_*ST*_ values for the ISL/NOR, ISL/SWE, ISL/DNK and ISL/FIN ranged from 0.103 to 0.133 (highest). Overall, the lowest *F*_*ST*_ value was found between the SWE- and FIN-subpopulations and the highest between the ISL- and DNK-subpopulations (Table 1).

**Table 1:** Pairwise correlation of the fixation index or *F*_*ST*_ values between subpopulations of rutabaga accessions from Denmark, Finland, Iceland, Norway and Sweden

	DNK	FIN	ISL	NOR	SWE
DNK	0.000				
FIN	0.088	0.000			
ISL	0.133	0.124	0.000		
NOR	0.067	0.067	0.103	0.000	
SWE	0.050	0.032	0.106	0.042	0.000

*F*_*ST*_ values between subpopulations ; *DNK* Denmark, *FIN* Finland, *ISL* Iceland, *NOR* Norway, *SWE* Sweden

**Fig. 3:**
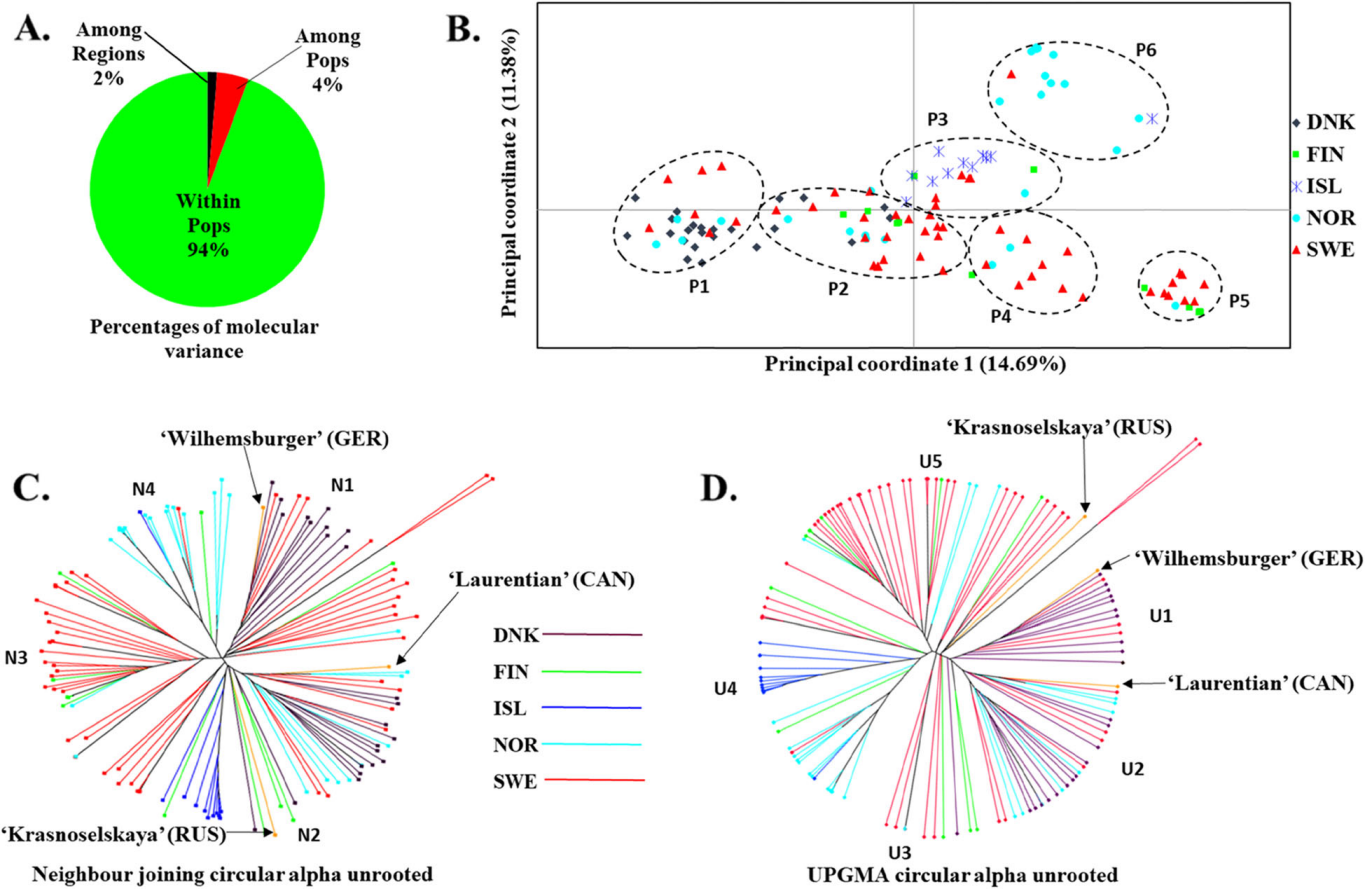
Analysis of molecular variance (AMOVA) partitioning of molecular variance among regions, populations and within accessions **(A)**. Principal coordinates analysis (PCoA) **(B)**, Neighbour joining (NJ) **(C)**, and Unweighted pair group method with arithmetic mean (UPGMA) **(D)** analyses with 6861 SNP markers grouped the 124 rutabaga accessions from Norway, Sweden, Finland, Denmark and Iceland into 6, 4 and 5 subgroups, respectively. The positions of the three out-groups, Laurentian (CAN), Wilhemsburger (GER) and Krasnoselskaya (RUS), are indicated on the NJ and the UPGMA trees

The analysis of molecular variance (AMOVA) of the distance matrices obtained with TASSEL (Trait Analysis by aSSociation, Evolution and Linkage) and GenAlEx software for the rutabaga accessions were highly correlated (Tables S2a and S2b). The AMOVA among and within the five populations partitioned the overall genetic variance into three parts: ≈ 94% attributable to within population differences, whereas ≈ 5% and ≈ 1% of the variation occurred among populations and among regions, respectively (*P* = 0.108) (Fig. 3A). This suggested only minor differences in the entire rutabaga populations from the different countries.

Pairwise comparison of the AMOVA (Φ_PT_) between the populations, however, revealed a higher genetic variance (18 to 27%) between the ISL-subpopulation and the NOR-, SWE-, FIN- and DNK-subpopulations (Table 2). Furthermore, the rutabaga accessions from Iceland and Denmark were the most genetically diverse (Φ_PT_ = 27%), followed by accessions from Iceland and Finland (Φ_PT_ = 24%). In contrast, rutabaga accessions from Sweden and Finland were the most similar (Φ_PT_ = 2%) followed by accessions from Norway and Sweden (Φ_PT_ = 7%).

**Table 2:** Pairwise comparison between population genetic variance of 124 rutabaga accessions from Denmark, Finland, Iceland, Norway and Sweden

	DNK	FIN	ISL	NOR	SWE
DNK	–				
FIN	16%	–			
ISL	27%	24%	–		
NOR	14%	12%	21%	–	
SWE	9%	2%	18%	7%	–

Values indicate genetic variance between populations

*DNK* Denmark, *FIN* Finland, *ISL* Iceland, *NOR* Norway, *SWE* Sweden

Thus, the vast majority of the genetic variability could be attributed to within population differences. Nevertheless, the pairwise comparison of the subpopulations suggested that considerable variation existed between the rutabagas from the different countries.

### Cluster analyses

The principal coordinate analysis (PCoA) based on the 6861 SNP markers clustered the 124 rutabaga accessions into six heterogeneous subgroups (Fig. 3B) using the first (PCoA1 ≈ 14.7% of genetic variance) and second (PCoA2 ≈ 11.4% of genetic variance) principal coordinates. Clearly, the rutabaga accessions from Sweden, Norway and Finland were distributed across almost all of the subgroups (P1 to P6 in Fig. 3B). In contrast, the accessions from Iceland and Denmark were concentrated in subgroup P3 and subgroups P1 and P2, respectively (Fig. 3B).

The neighbour-joining (NJ) based on the 6861 SNP markers clustered the 124 rutabaga accessions into four major branches (Fig. 3C). The unrooted phylogenetic trees indicated that the accessions from Sweden were distributed into three of the branches (N1, N2 and N3), those from Norway, Finland and Denmark were segregated into two of the branches (N2 and N4, N2 and N3 and N1 and N2, respectively), whereas accessions from Iceland were concentrated in one branch (N2) (Fig. 3C).

The unweighted pair group method with arithmetic mean (UPGMA) based on the 6861 SNP markers indicated that the trees for the 124 rutabaga accessions were clustered into five major branches (Fig. 3D). The accessions from Sweden, Norway, and Finland were widely distributed across at least four of the major branches (Fig. 3D). Similar to the branching patterns in the NJ analysis, the rutabaga accessions from Denmark and Iceland clustered into two branches (U1 and U2) or one branch (U4), respectively.

Overall, the three multivariate analyses (PCoA + NJ + UPGMA) suggested the existence of four to six groups in the rutabaga accessions. However, correlations with their geographic origin were very low, except for the accessions from Iceland.

The unrooted trees used to depict the NJ and UPGMA do not imply a known ancestral root of the three out-groups (which are coloured orange in Fig. 3C and D). However, the results suggested that the rutabaga ‘Wilhemsburger’ was in the first branch (N1 of the NJ unrooted tree), while ‘Laurentian’ and ‘Krasnoselskaya’ both were grouped in the second branch (N2 of the NJ unrooted tree) (Fig. 3C). In the case of the unrooted tree used to depict UPGMA, ‘Wilhemsburger’, ‘Laurentian’ and ‘Krasnoselskaya’ were grouped in the first (U1), second (U2) and fifth branch (U5), respectively (Fig. 3D).

The NJ and UPGMA representation of the similarity matrices as a phylogram (Figs. S1a and S1b) and a circular rooted (Figs. S2a and S2b) diagram are included in the Supplementary Materials. These indicate even closer groupings of the accessions based on their geographic origins.

### Bayesian population structure analysis

The *STRUCTURE* analysis was run 11 times with the accessions unassigned and 11 times with the accessions assigned to their respective countries of origin. Table 3 summarizes the *STRUCTURE* results used to infer the population genetic structure of the rutabaga accessions from the Nordic countries. The number of clusters (*K*) determined following the method of Evanno et al. [34] indicated *∆K* statistic values of *K* = 2 to 9, while the four alternative statistics (MedMedK, MedMeaK, MaxMedK and MaxMeaK) determined following Puechmaille [35] and Li and Liu [36] indicated 3 to 4 clusters (Table 3). Increasing the number of replications from 10 to 20 produced cluster numbers similar to the above. These suggested that the Puechmaille [35] and Li and Liu [36] method was more consistent than the Evanno et al. [34] method for inferring the population genetic structure of the rutabaga accessions from the Nordic countries. Based on the *∆K* statistic values, there was no significant difference in *STRUCTURE* run # 1, 2, 3 and 8 for analysis done with the accessions unassigned and for analysis with the accessions assigned to their respective countries of origin. In contrast, significant differences were found for *STRUCTURE* run # 4, 5, 6, 9, 10 and 11. The two methods produced approximately the same number of clusters (*K* = 3 to 4) at Burn-in and MCMC lengths each of 50,000 and at *K* = 1–10 and for 10 replicates (i.e. run #7) (Table 3).

**Table 3:** Determination of the number of cluster sets in 124 rutabaga accessions from Denmark, Finland, Iceland, Norway and Sweden using the Evanno et al. (2005) and Puechmaille et al. (2016) methods

Structure	Burn-in lengths	MCMC^*^ lengths	Number of clusters (*K*)	Number of Reps	Number of populations^α^		Number of Populations^β^			
ran #					∆K (Unassigned)^a^	∆K (Assigned)^b^	MedMedK	MedMeaK	MaxMedK	MaxMeaK
1	5000	5000	10	10	9	8	3	4	4	4
2	5000	5000	10	20	8	8	3	4	4	4
3	10000	10000	10	10	9	8	3	4	4	4
4	10000	10000	10	20	2	9	3	4	4	4
5	20000	20000	10	10	8	2	3	4	4	4
6	20000	50000	10	10	9	2	3	4	4	4
7	50000	50000	10	10	3	3	3	4	4	4
8	10000	100000	10	10	8	6	3	4	4	4
9	20000	100000	10	10	2	9	3	4	4	4
10	50000	100000	10	10	9	2	3	4	4	4
11	100000	100000	10	10	3	9	3	4	4	4

^*^MCMC Markov Chain Monte Carlo

^α^ The ad hoc *∆K* method (Evanno et al. 2005); ^a^ Accessions unassigned to any population or country; ^b^ Accessions assigned to their countries of origin

^β^ The median (MedMedK and MaxMedK) or mean (MedMeaK and MaxMeaK) estimators used to determine which subpopulations belonged to a cluster (*K*) (Puechmaille et al. 2016)

**Fig. 4:**
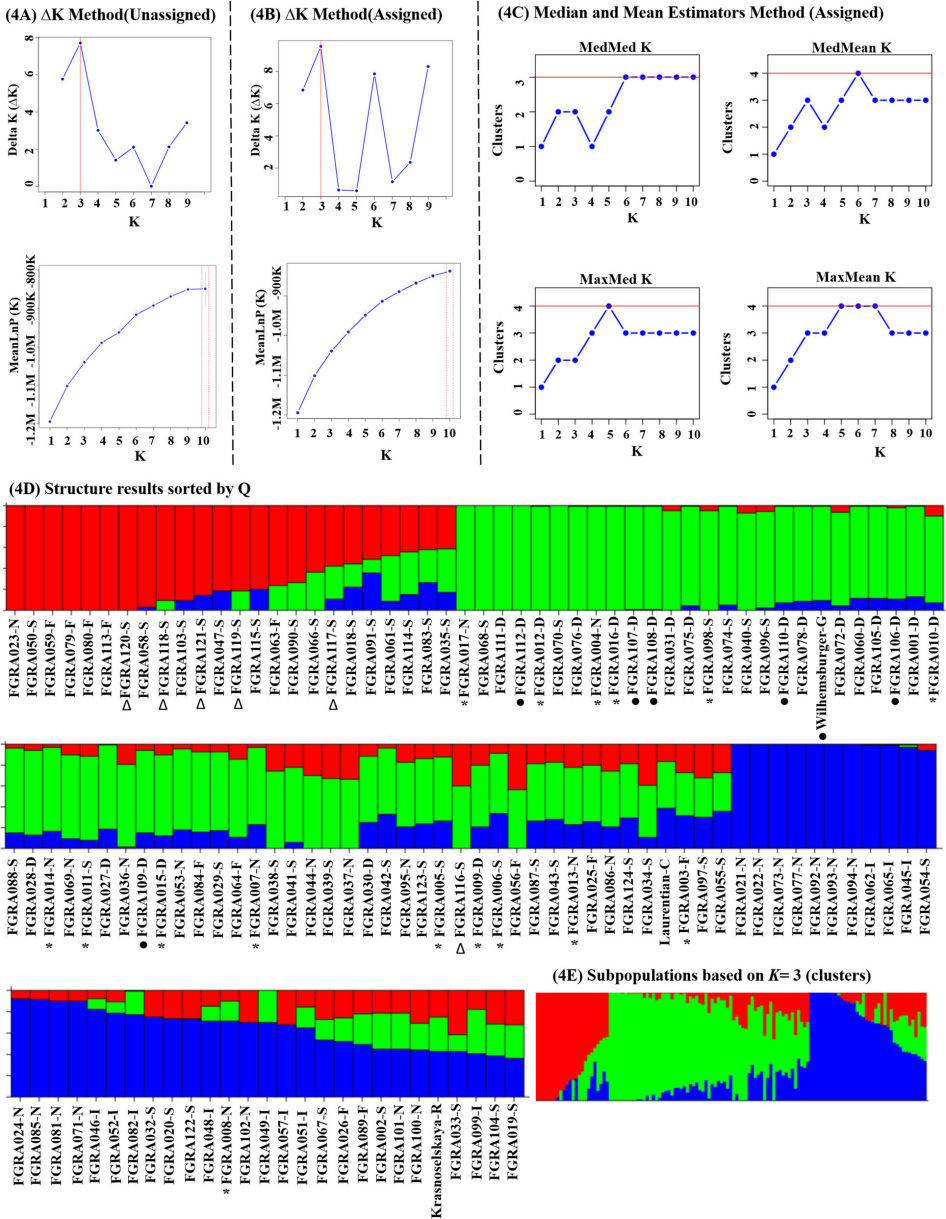
Bayesian cluster analysis of 124 rutabaga accessions from Norway, Sweden, Finland, Denmark and Iceland estimated using the software *STRUCTURE* based on 6861 SNP markers. The optimal value of *K*, determined by the method of Evanno et al. (48) with populations unassigned **(A)** or assigned **(B)** to their respective countries, as well as by the method of Puechmaille [37] and Li and Liu [38] **(C)**, suggested that the 124 rutabaga accessions could be placed into 3 or 4 clusters (*K* = 3 most likely). Detailed Bayesian clustering of 124 rutabaga accessions by the CLUMPAK program [39] **(D-E)**. Each column or rectangular bar represents the individual rutabaga accessions used in this study, while each colour represents one gene pool and the stacked bars with different colours represent admixtures with their shared ancestry components **(D**). Simplified view suggests three ancestral populations (**E**)

Plots of MedMedK, MedMeaK, MaxMedK and MaxMeaK as well as log-likelihood (*lnK*) against the number of clusters suggested the presence of subpopulations in the accessions (Fig. 4 and S3). Based on a threshold for similarity score of 70%, 66.1% of the accessions were placed into one of the three clusters while 33.9% were classified as admixtures (Table 4). Excluding the admixture, 91.3% of the accessions from Denmark and 72.7% of the accessions from Iceland were present in only one cluster (1 and 2, respectively). In contrast, 58.3% of the accessions from Finland and 42.0% of the accessions from Sweden were present in clusters 1 and 3, while 75.0% of the accessions from Norway were present in clusters 1 and 2 (Table 4). The German rutabaga ‘Wilhemsburger’ was placed in cluster 1 along with some of the accessions from Denmark, Finland, Norway and Sweden. The Canadian rutabaga ‘Laurentian’ and the Russian rutabaga ‘Krasnoselskaya’ were admixtures. Overall, the number of clusters (3 to 4) obtained in the *STRUCTURE* analysis with the Puechmaille [35] and Li and Liu [36] method was consistent and comparable with the 4–6 subgroups obtained in the multivariate analysis. In contrast, the number of clusters determined following Evanno et al. [34] were not consistent and varied widely.

**Table 4:** Inferred ancestry of 124 rutabaga accessions from Denmark, Finland, Iceland, Norway and Sweden based on membership coefficients

Cluster	Origin of rutabaga^α^					Total Number	Ave genetic Distance within subgroup^β^
(*K*)	DNK (23)	FIN (12)	ISL (11)	NOR (28)	SWE (50)		
1^*^	21	2	0	8	11	42	0.1805
2	0	0	8	13	4	25	0.4218
3	0	5	0	1	10	16	0.2245
Admixture^†♠^	2	5	3	6	25	41	
In-Cluster	91.3%	58.3%	72.7%	78.6%	50.0%	66.9%	
Admixture	8.7%	41.7%	27.3%	21.4%	50.0%	33.1%	

^α^ Sample sizes of the Accessions were assigned to a specific cluster (*K*) if *P* ≥ 0.70 and those that did not meet this threshold were considered as admixture

Placement of out-groups:*‘Wilhemsburger’ (Germany) = *K*1 and ^†^‘Laurentian’ (Canada) and ^♠^ ‘Krasnoselskaya’ (Russia) = Admixture

^β^The average genetic distance within each cluster or subgroup was obtained from the *STRUCTURE* results

### Clustering of genotypes with similar names

The NJ, UPGMA and *STRUCTURE* analyses placed the majority of the accessions with similar names but with different accession numbers into the same cluster, irrespective of their countries of origin. For example, the three analyses placed all six ‘Wilhemsburger’ accessions (FGRA112D, FGRA107D, FGRA108D, FGRA110D, FGRA106D and FGRA109D) in the same cluster as ‘Wilhemsburger’ from Germany, which was used as an out-group (Fig. 4D and S2). Similarly, the NJ and UPGMA analyses placed all six (FGRA120S, FGRA118S, FGRA121S, FGRA119S, and FGRA117S) ‘Östgota’ accessions into one group (Fig. S2), while the *STRUCTURE* analysis placed five of the six into one group (except FGRA116S) (Fig. 4D). In the case of ‘Bangholm’ accessions, both NJ and UPGMA captured 13 of the 16 accessions into one group, while the remaining three accessions (FGRA 003, FGRA011 and FGRA008) were placed into two groups (Fig. S2). The *STRUCTURE* analysis placed 15 of the 16 ‘Bangholm’ accessions (except FGRA008) in the same cluster (Fig. 4D). Therefore, the clustering of the rutabaga accessions using NJ, UPGMA and *STRUCTURE* analyses was very consistent.

## Discussion

A comprehensive body of literature exists on rutabagas in the main Nordic languages (Personal communication, Prof. Ann-Charlotte Wallenhammar, Swedish University of Agricultural Sciences). This probably reflects the transmission of seeds and information on agronomic practices for rutabaga cultivation in the Nordic region since medieval times [4]. Turesson [40–42] observed that when the same species of plants were grown in different habitats over many years, they differed from each other in stature, colour, morphology and texture of leaves, stem, flowers and seed. Consequently, rutabagas that are adapted to different climatic and geographic environments will develop different morphological traits.

In this study, SNP markers and combinations of allele- and distance-based population genetics statistics, multivariate clustering and Bayesian methods were used to examine genetic diversity and differentiation in rutabaga accessions from Norway, Sweden, Finland and Denmark and Iceland. Diers and Osborn [32] used rutabaga accessions as an out-group in genetic diversity studies of *B. napus*, whereas Mailer et al. [33] and Bus et al. [31] compared rutabagas with spring oilseed rape, winter oilseed rape, fodder and vegetable types. Fewer than 100 SSR, RFLP and RAPD markers, however, were used in those studies compared with the 6861 SNP markers in the current study. In contrast, Gazave et al. [28] and Zhou et al. [27] identified 1,081,925 and 1,197,282 SNP markers using an Illumina Hiseq single-end sequencing and Specific-Locus Amplified Fragment sequencing (SLAF-Seq), respectively. Similarly, An et al. [29] and Lu et al. [30] obtained 372,546 and 675,457 high-quality SNPs by RNA-sequencing, respectively. The four studies used over 30,000 SNP markers for genetic structure analysis, which is ≈ 4 × the 6861 markers used in our study. In general, the use of more markers improves the accuracy of subpopulation clustering. Moreover, DNA sequencing can provide information on gene-rich regions and is particularly useful for species with limited or no genome information. Nonetheless, DNA sequencing is more costly than the use of array-based SNP markers, although extensive validation of the SNP markers is needed before they can be used for genetic diversity studies.

The average of 2.012 ± 0.003 alleles per SNP locus obtained in this study was less than the 4.78 alleles per SSR locus found by Bus et al. [31]. This was expected, since SSR markers are multi-allelic codominant markers while SNP markers are often bi-allelic. The mean expected heterozygosity ($\overline{H}$e) or gene diversity (D) of 0.283 ± 0.001 obtained in this study was less than the 0.43 reported by Bus et al. [31], likely because the 73 accessions examined in the latter represented a more diverse collection from 19 countries with a much wider geographical distribution (Europe, North America, Asia, New Zealand and the North Africa). The lower allelic diversity summary statistics with SNP markers compared with SSR markers also has been reported in rice [43, 44], barley [45], mushrooms [46] and other species. An et al. [29] reported SNP density of 0.286–1.080 per kb. The SNP density in this study ranged from 0.009 to 0.184 kb per chromosome [12]. This is a reflection of the higher numbers of SNPs detected by genome resequencing methods.

The pairwise fixation index (*F*_ST_) obtained in the current study ranged from 0.032 to 0.133, which was within the 0.054 reported by Bus et al. [31]. Therefore, the use of SNP and SSR markers confirmed that genetic differentiation in the rutabaga accessions is low and there is a high degree of genetic exchange within these accessions. The observation in earlier studies that rutabagas clustered separately from spring, winter, fodder and vegetable *Brassica* species [31–33] could be due to many rutabagas being landraces with different morphological adaptions to various geographic and climatic regions. The significantly higher pairwise *F*_ST_ values for ISL-DNK (0.133), ISL-FIN (0.124), ISL-SWE (0.106) and ISL-NOR (0.103) compared with the pairwise *F*_ST_ values for DNK, FIN, NOR and SWE (range 0.032 to 0.088) may reflect enrichment caused by lack of mating between the Icelandic sub-population and the rest of the subpopulations. As an island in the North Atlantic, Iceland is geographically isolated from the other Nordic countries. This isolation, combined with possible differences in microclimatic and soil conditions, may have resulted in less exchange of germplasm between Iceland and Norway, Denmark, Finland and Sweden.

The hierarchical clustering of the genotype data by the use of NJ, UPGMA and PCoA analyses yielded 4 to 6 subgroups in the rutabaga accessions. Previous studies [35, 47] reported that the *STRUCTURE* program did not reliably identify the main clusters within a subpopulation. Therefore, in this study, many runs of the *STRUCTURE* program were carried out to obtain convergence before the ‘best’ number of clusters was determined. In addition, we compared the number of inferred clusters from the alternative statistics, MedMedK, MedMeaK, MaxMedK and MaxMeaK [35] to the ∆K method [34] to decide the ‘best’ number of clusters. The possible number of clusters was 2, 3, 4, 6, 8 or 9, with the ‘best’ or most consistent of the two methods being 3 to 4. The variable numbers of clusters in the PCoA, UPGMA, NJ and *STRUCTURE* analyses could be due to the low levels of genetic variability and the high genetic exchange among rutabaga accessions from the Nordic countries. This was confirmed further by the high degree (33.1%) of admixtures detected by the Bayesian population structure analyses. Inspection of clusters obtained by the use of the four genetic structure analysis methods showed that the majority (90–100%) of accessions with similar names were in the same group irrespective of their countries of origin. This suggests that the 6861 SNP markers were able to reliably determine the number of clusters in the 124 rutabaga accessions from the Nordic countries. The population structure analyses were in agreement with the allele diversity summary statistics obtained in this study.

## Conclusion

Our results showed that the majority of the genetic differences in rutabaga accessions from the Nordic countries were present within the Icelandic subpopulations, while accessions from Norway, Sweden, Finland and Denmark were genetically very similar. Given these findings, based on molecular genetics analyses, there may be value in additional and more detailed study of the morphological traits of accessions originating from these different countries. Lastly, these rutabaga accessions should be of great interest to breeders for increasing genetic diversity in *B. napus*.

## Materials and methods

### Plant material

One-hundred twenty-four rutabaga accessions collected from the Nordic Genetic Resource Center and used for genome wide association studies by Fredua-Agyeman et al. (12) were included in this study. These consisted of 23 accessions from Denmark, 12 from Finland, 11 from Iceland, 28 from Norway and 50 accessions from Sweden (Fig. 1). Hereafter, the rutabaga accessions from the five countries will be referred to as the DNK-, FIN-, ISL-, NOR- and SWE-subpopulations, respectively. In addition, seeds of three commercial rutabaga cultivars, ‘Laurentian’ from Canada, ‘Wilhemsburger’ from Germany and ‘Krasnoselskaya’ from Russia, were included as the out-group. Details on the accessions are presented in Table S3. Two to four seeds of each accession were grown in 13 × 13 × 15 cm pots filled with Sunshine Mix #4 Aggregate Plus Growing Mix (Sungro Horticulture Canada Ltd) and kept in a growth chamber with a 16 h/8 h (22 °C) day/night cycle for 4 weeks. Leaf tissue (~ 0.25 g) was collected from two plants of each accession in 1.5 mL microcentrifuge tubes on ice. The samples were stored at − 20 °C and shipped on dry ice for SNP genotyping.

### SNP genotyping and filtering

SNP genotyping of the 124 rutabaga accessions and three commercial cultivars was performed with a 15 K SNP *Brassica* array at TraitGenetics GmbH, Gatersleben, Germany. These markers were part of 34,248 A and C genome-specific and polymorphic SNP markers identified during an analysis of 432 *B. napus* and ancestral diploid genotypes by Clarke et al. [25]. The majority of the SNP assays targeted single loci within the genome to limit the impact of genome duplication and to simplify SNP calling [25]. The SNP marker positions were mapped to the A genome of *B. rapa* [48], the C genome of *B. oleracea* [49] and the A and C genomes of *B. napus* [50]. The majority of the SNP loci were found in non-coding regions [27].

After genotyping, filtering was done to remove monomorphic and low coverage site SNP markers, those with minor allele frequency (MAF) < 0.05, and SNPs with missing data for > 5% of the accessions. Six thousand eight hundred sixty-one SNP markers were retained for the calculation of the genetic diversity indices and the population structure analyses. These comprised 4390 A-genome and 2471 C-genome SNP markers distributed across all 19 chromosomes of *B. napus* [12].

### Allele frequency-based population structure analyses

The proportion of polymorphic loci (%*P*), the mean number of alleles per locus (*Na*), the mean number of effective alleles per locus (*Ne*), the mean expected heterozygosity ($\overline{H}$_*e*_), the mean unbiased expected heterozygosity (*U* $\overline{H}$_*e*_), the mean number of alleles with a frequency ≥ 5% (*Na Freq* ≥ 5%), mean number of common alleles found in ≤25% and ≤ 50% of the subpopulations (*Na comm* ≤ 25% and *Na comm* ≤ 50%; respectively) and Shannon’s information index (*I*), within and among subpopulations, as well as Wright’s [51] genetic differentiation F-statistics (*F*_*ST*_) between the populations were determined with GenAlEx 6.5 [37, 52]. The *F*_ST_ values were assessed at 1000 random permutations across the 6861 loci.

In addition, the polymorphism information content (PIC), minor allele frequency (MAF) and the expected heterozygosity at any given locus (*He*) also called gene diversity (*D*) [38] were evaluated for the DNK-, FIN-, ISL-, NOR- and SWE-subpopulations and the entire population using POWERMAKER v3.25 [39].

### Distance-based population structure analyses

The genetic and similarity distance matrices within and among the subpopulations were calculated for the 6861 SNP markers and the 124 accessions using both GenAlEx 6.5 [37, 52] and TASSEL v5.2.2.5 [53].

The matrices were used to test the hierarchical partitioning of the analysis of molecular variance (AMOVA) among regions, populations and within accessions and their level of statistical significance was assessed based on 10,000 permutations [54]. In addition, patterns in the population were inferred or visualized by principal coordinates analysis (PCoA) [55]. The AMOVA and PCoA were conducted with GenAlEx 6.5. The unweighted pair group method with arithmetic mean (UPGMA) [56] and neighbour-joining (NJ) [57] clustering methods implemented in TASSEL v5.2.2.5 [53] were used to generate phylogenetic trees.

### Bayesian population structure analyses

A Bayesian clustering approach, applying a Markov Chain Monte Carlo (MCMC) algorithm implemented in the population-genetic software *STRUCTURE* v2.3.4 [58], was used to assign the 124 rutabaga accessions from the various countries into a number of genetically homogeneous clusters (*K*) based on the 6861 SNP markers. In addition, *STRUCTURE* was used to assign the rutabagas ‘Laurentian’ (Canada), ‘Wilhemsburger’ (Germany) and ‘Krasnoselskaya’ (Russia) into the Nordic subpopulations showing similar variation patterns.

*STRUCTURE* was run using the admixture model with correlated allele frequencies at a series of burn-in lengths from 5000 to 100,000 iterations and MCMC run lengths from 5000 to 100,000 permutations. In addition, *STRUCTURE* was run with the accessions unassigned to any population or country and with the accessions assigned to their countries of origin. Runs for each *K* = 1–10 were replicated 10 or 20 times. These were done to determine the parameters needed to reach convergence. The most likely number of clusters (the ad hoc *∆K* test) and average log-likelihood plots were determined following Evanno et al. [34] with STRUCTURE HARVESTER v0.6.94 [59]. Secondly, the median (MedMedK and MaxMedK) or mean (MedMeaK and MaxMeaK) estimators of the “best” *K* were used to group subpopulations into clusters with STRUCTURESELECTOR [35, 36]. Accessions were assigned to a specific cluster if the probability of membership was ≥0.70, with those that did not meet this threshold considered as an admixture.

### Statistical analysis

Statistical significance between means of the parameters (pairwise and overall) was established by Fisher’s protected least significant difference (LSD) test (*P* ≤ 0.05) using SAS v. 9.4 (SAS Institute, Inc., Cary, NC, USA).

## Supplementary Information

**Additional file 1.**

## Data Availability

The accession numbers of the rutabaga genotypes used in the current study are provided in Table S3 of the supplementary materials. In addition, the datasets generated during the current study are available in the manuscript or the supplementary materials. The SNPs used for genotyping are available from Clark et al. [51] or can be downloaded at https://static-content.springer.com/esm/art%3A10.1007%2Fs00122-016-2746-7/MediaObjects/122_2016_2746_MOESM3_ESM.pdf. Any other information can be obtained from the corresponding author.

## Abbreviations

PIC: Polymorphism information content
%*P*: The proportion of polymorphic loci
*Na*: The mean number of alleles per locus
*Ne*: The mean number of effective alleles per locus
$\overline{H}$_*e*_: The mean expected heterozygosity
*U*$\overline{H}$_*e*_: The mean unbiased expected heterozygosity
*Na Freq* ≥ 5%: The mean number of alleles with a frequency ≥ 5%
*Na comm* ≤ 25% and *Na comm* ≤ 50%: Mean number of common alleles found in ≤25% and ≤ 50% of the subpopulations, respectively
*I*: Shannon’s information index
*F*_*ST*_: Wright’s genetic differentiation F-statistics
MAF: Minor allele frequency
D: Gene Diversity
TASSEL: Trait analysis by association evolution and linkage
MCMC: Markov Chain Monte Carlo
AMOVA: Analysis of molecular variance
PCoA: Principal coordinate analysis
UPGMA: The unweighted pair group method with arithmetic mean
NJ: Neighbour-joining
