# Supplementary material for: Molecular genetic diversity and population structure analyses of rutabaga accessions from Nordic countries as revealed by single nucleotide polymorphism markers
Source: BMC Genomics. 2021 Jun 12;22:442. doi: 10.1186/s12864-021-07762-4 (PMC8199374; doi:10.1186/s12864-021-07762-4)
Supplement: Supplementary file 1 — Additional file 1. [file 12864_2021_7762_MOESM1_ESM.pdf]

Molecular genetic diversity and population structure analyses of rutabaga accessions from Nordic countries as revealed by single nucleotide polymorphism markers

Zhiyu Yu[*], Rudolph Fredua-Agyeman[*], Sheau-Fang Hwang, Stephen E. Strelkov[¥]

Department of Agricultural, Food and Nutritional Science, University of Alberta, Edmonton, AB, T6G 2P5, Canada.

[¥] **Correspondence:**

Stephen E. Strelkov

E-mail: strelkov@ualberta.ca

[*] These two authors contributed equally to this work

Supplementary Materials

Table S1. Marker information and genetic diversity statistics (mean ± SE) for 124 rutabaga accessions from Denmark, Finland, Iceland, Norway and Sweden using 6861 SNP markers.

Pop	N	%P	N_a	N_e	I	H_e	UH_e
Denmark	23	93.8	2.117 ± 0.006	1.515 ± 0.004	0.474 ± 0.003	0.301 ± 0.002	0.308 ± 0.002
Finland	12	88.5	1.940 ± 0.005	1.484 ± 0.004	0.445 ± 0.003	0.288 ± 0.002	0.301 ± 0.002
Iceland	11	67.9	1.707 ± 0.006	1.299 ± 0.004	0.305 ± 0.003	0.191 ± 0.002	0.200 ± 0.002
Norway	28	95.3	2.063 ± 0.005	1.488 ± 0.004	0.456 ± 0.003	0.292 ± 0.002	0.297 ± 0.002
Sweden	50	99.6	2.236 ± 0.005	1.590 ± 0.004	0.535 ± 0.002	0.345 ± 0.002	0.348 ± 0.002
All	124	89.02 ± 5.57	2.012 ± 0.003	1.475 ± 0.002	0.443 ± 0.001	0.283 ± 0.001	0.291± 0.001

N = sample size; *%P* = percentage of polymorphic loci; N_a = mean number of alleles; N_e = number of effective alleles; *I* = Shannon's information index; H_e = expected heterozygosity and UH_e = unbiased expected heterozygosity.

Table S2a. Analysis of molecular variance (AMOVA) among and within 124 rutabaga accessions from Denmark, Finland, Iceland, Norway and Sweden based on genotypic distance matrix with the software GenAlEx.

Source of variance	DF	SS	MS	Estimated Variance	% Total	*P*
Among regions	2	19086.56	9543.28	59.75	1.4%	0.0214
Among populations	2	18447.25	9223.63	200.90	4.7%	0.0001
Within populations	119	477078.32	4009.06	4009.06	93.9%	0.0001

DF = Degree of freedom; SS = Sum of squares; MS = mean squared deviation. The probability is based on standard permutation across the full data set. The codon-genotypic option in GenAlEx, under the assumption of independence, was used to calculate sets of squared genetic distances summed across all loci.

Table S2b. Analysis of molecular variance (AMOVA) among and within 124 rutabaga accessions from Denmark, Finland, Iceland, Norway and Sweden based on similarity matrix calculated with the software TASSEL.

Source of variance	DF	SS	MS	Estimated Variance	% Total	*P*
Among regions	2	0.735	0.367	0.002	1.4%	0.0201
Among populations	2	0.706	0.353	0.007	4.3%	0.0001
Within populations	119	19.176	0.161	0.161	94.3%	0.0001

DF = Degree of freedom; SS = Sum of squares; MS = mean squared deviation. The probability is based on standard permutation across the full data set. TASSEL calculates distance as 1 - IBS (identity by state) similarity, where IBS is defined as the probability that alleles drawn at random from two individuals at the same locus are the same.

Table S3. List of 124 rutabaga accessions from five Nordic countries (Denmark, Finland, Iceland, Norway and Sweden) included in this study of genetic diversity.

MS Identifier	Accession	Common Name	Origin
FGRA001-D	NGB20826	AMERICAN PURPLE TOP	Denmark
FGRA009-D	NGB13795	BANGHOLM HINDERUPGAARD 9	Denmark
FGRA010-D	NGB13806	BANGHOLM HUNSBALLE 9	Denmark
FGRA012-D	NGB8372	BANGHOLM PAJBJERG REGENT	Denmark
FGRA015-D	NGB13812	BANGHOLM WIBOLTT	Denmark
FGRA016-D	NGB13813	BANGHOLM WILBY ØTOFTE	Denmark
FGRA027-D	NGB1610	DALO TRIFOLIUM	Denmark
FGRA028-D	NGB4128	DIMA TRIFOLIUM	Denmark
FGRA030-D	NGB13816	DÆHNFELDT	Denmark
FGRA031-D	NGB1611	FAMA DÆHNFELDT	Denmark
FGRA060-D	NGB1612	MAGRES PAJBJERG	Denmark
FGRA072-D	NGB13801	RECORD TASHUPGAARD	Denmark
FGRA075-D	NGB1613	RUTA ØTOFTE	Denmark
FGRA076-D	NGB8373	SAHNA PAJBJERG	Denmark
FGRA078-D	NGB1614	SATOR ØTOFTE	Denmark
FGRA105-D	NGB1615	WILBY ØTOFTE	Denmark
FGRA106-D	NGB13798	WILHELMSBUGER	Denmark
FGRA107-D	NGB13804	WILHELMSBURGER DAENO 9	Denmark
FGRA108-D	NGB8384	WILHELMSBURGER, DANILA	Denmark
FGRA109-D	NGB20827	WILHELMSBURGER DANILA TRIFOLIUM	Denmark
FGRA110-D	NGB8385	WILHELMSBURGER, REFORM	Denmark
FGRA111-D	NGB13815	WILHELMSBURGER TRIFOLIUM 9	Denmark
FGRA112-D	NGB13814	WILHELMSBURGER ØTOFTE 9	Denmark
FGRA003-F	NGB23908	BANGHOLM	Finland
FGRA025-F	NGB13791	BRYUKVA	Finland
FGRA026-F	NGB13808	BRYUKVA	Finland
FGRA056-F	NGB4408	LAITIALA AP0106	Finland
FGRA059-F	NGB4409	LYTTYLÄ AP0101	Finland
FGRA063-F	NGB24437	MESSUKYLÄ	Finland
FGRA064-F	NGB13792	MUSTIALA	Finland
FGRA079-F	NGB24436	SIMO (SIMO 1)	Finland
FGRA080-F	NGB14152	SIMO (SIMO 2)	Finland
FGRA084-F	NGB20823	SORTAVALA	Finland
FGRA089-F	NGB13805	TAMMISTO	Finland
FGRA113-F	NGB1176	VILLALA ME0101	Finland
FGRA045-I	NGB9915	HNAUSAROFA	Iceland
FGRA046-I	NGB9911	HVAMMSROFA	Iceland
FGRA048-I	NGB20824	KAFIFAFELLSROFUR	Iceland
FGRA049-I	NGB4142	KALFAFELLSROFA	Iceland
FGRA051-I	NGB9909	KOLSHOLTSHELLISROFA	Iceland

FGRA052-I	NGB9916	KORPUROFA	Iceland
FGRA057-I	NGB9914	LAUGABOLSROFA	Iceland
FGRA062-I	NGB9910	MARIUBAKKAROFA	Iceland
FGRA065-I	NGB9913	MÖGGUROFA	Iceland
FGRA082-I	NGB9907	SLETTUROFA	Iceland
FGRA99-I	NGB13811	VESTMANNAEYRA	Iceland
FGRA004-N	NGB13800	BANGHOLM ELITE	Norway
FGRA007-N	NGB7793	BANGHOLM GOKSTAD	Norway
FGRA008-N	NGB2657	BANGHOLM HAUKEBØ ST	Norway
FGRA013-N	NGB4568	BANGHOLM SANDNES	Norway
FGRA014-N	NGB4567	BANGHOLM VEREIDE	Norway
FGRA017-N	NGB10657	BANGHOLM WILBY ØTOFTE II	Norway
FGRA021-N	NGB5015	BRANDHAUG	Norway
FGRA022-N	NGB522	BRANDHAUG, MARKA STAMME	Norway
FGRA023-N	NGB5016	BREDIK	Norway
FGRA024-N	NGB7792	BRENDBERGLI	Norway
FGRA036-N	NGB13794	GRO	Norway
FGRA037-N	NGB11559	GRY	Norway
FGRA044-N	NGB9268	GØTA LEDAAL STAMME	Norway
FGRA053-N	NGB11558	KVIMAR	Norway
FGRA069-N	NGB7794	OLSGÅRD ELITE	Norway
FGRA071-N	NGB7795	RANAKÅLROT	Norway
FGRA073-N	NGB10000	REDY	Norway
FGRA077-N	NGB523	SALTA	Norway
FGRA081-N	NGB10656	SIMONETTE KVANDE	Norway
FGRA085-N	NGB5017	STENHAUG	Norway
FGRA086-N	NGB4133	STENHAUG	Norway
FGRA092-N	NGB9274	TRØNDERSK BRANDHAUG	Norway
FGRA093-N	NGB10659	TRØNDERSK HYLLA	Norway
FGRA094-N	NGB4569	TRØNDERSK KVITHAMAR	Norway
FGRA095-N	NGB11560	VALLDALSKÅLROT	Norway
FGRA100-N	NGB5018	VIGE	Norway
FGRA101-N	NGB4134	VIGE	Norway
FGRA102-N	NGB24906	VIGOD	Norway
FGRA002-S	NGB21723	BAGGENS	Sweden
FGRA005-S	NGB13793	BANGHOLM FENIX	Sweden
FGRA006-S	NGB7175	BANGHOLM FENIX	Sweden
FGRA011-S	NGB13797	BANGHOLM ORIGINAL	Sweden
FGRA018-S	NGB17916	Bjursås (Bortas Bertils kålrot)	Sweden
FGRA019-S	NGB13474	BJURSÅS	Sweden
FGRA020-S	NGB17910	BOLTJÄRN	Sweden
FGRA029-S	NGB13799	DROTTNING	Sweden
FGRA032-S	NGB17918	FARFAR	Sweden
FGRA033-S	NGB11748	FINNMARKENS VITA KÅLRÖTTER	Sweden

FGRA034-S	NGB13637	GLOBUS	Sweden
FGRA035-S	NGB13120	GLOBUS	Sweden
FGRA038-S	NGB13119	GULLÅKER III	Sweden
FGRA039-S	NGB11688	GULLÅKER III	Sweden
FGRA040-S	NGB13796	GUL SVENSK	Sweden
FGRA041-S	NGB13802	GUL SVENSK	Sweden
FGRA042-S	NGB13663	GUL SVENSK (from SESAM)	Sweden
FGRA043-S	NGB13818	GUL SVENSK SVALÖFS ORIGINAL	Sweden
FGRA047-S	NGB17914	JANNE	Sweden
FGRA050-S	NGB21676	KLINT KARIN	Sweden
FGRA054-S	NGB11742	KÅLROT FROM ASPÅS	Sweden
FGRA055-S	NGB11744	KÅLROT FROM TÄLLBERG	Sweden
FGRA058-S	NGB17905	LJUSTORP	Sweden
FGRA061-S	NGB13770	MARIEROKÅLROT	Sweden
FGRA066-S	NGB18050	NORJÖ	Sweden
FGRA067-S	NGB17909	NUSNÄS	Sweden
FGRA068-S	NGB13790	ODAL	Sweden
FGRA070-S	NGB13809	PATRIA	Sweden
FGRA074-S	NGB6791	ROTABAGGE, KÅLROT	Sweden
FGRA083-S	NGB17915	SOLLROT	Sweden
FGRA087-S	NGB20825	SVALÖF VICTORIA	Sweden
FGRA088-S	NGB13807	SVENSK	Sweden
FGRA090-S	NGB11689	TIFFANY	Sweden
FGRA091-S	NGB17908	TRUTSGÅRD	Sweden
FGRA096-S	NGB20822	WEIBULLS FODDER	Sweden
FGRA097-S	NGB23910	WEIBULLS ORGINAL BALDER	Sweden
FGRA098-S	NGB13810	WEIBULLS ORIGINAL BANGHOLM	Sweden
FGRA103-S	NGB17906	VIKSJÖ	Sweden
FGRA104-S	NGB7176	VIKTORIA	Sweden
FGRA114-S	NGB17911	VINTJÄRN	Sweden
FGRA115-S	NGB17913	ÖSTERGYLLEN	Sweden
FGRA116-S	NGB13803	ÖSTGÖTA	Sweden
FGRA117-S	NGB23909	ÖSTGÖTA II	Sweden
FGRA118-S	NGB13638	ÖSTGÖTA II	Sweden
FGRA119-S	NGB10658	ÖSTGÖTA II	Sweden
FGRA120-S	NGB13118	ÖSTGÖTA II	Sweden
FGRA121-S	NGB11164	ÖSTGÖTA II	Sweden
FGRA122-S	NGB17917	ÖSTNOR	Sweden
FGRA123-S	NGB10667	DELTA	Sweden
FGRA124-S	NGB10668	SIGMA	Sweden

31

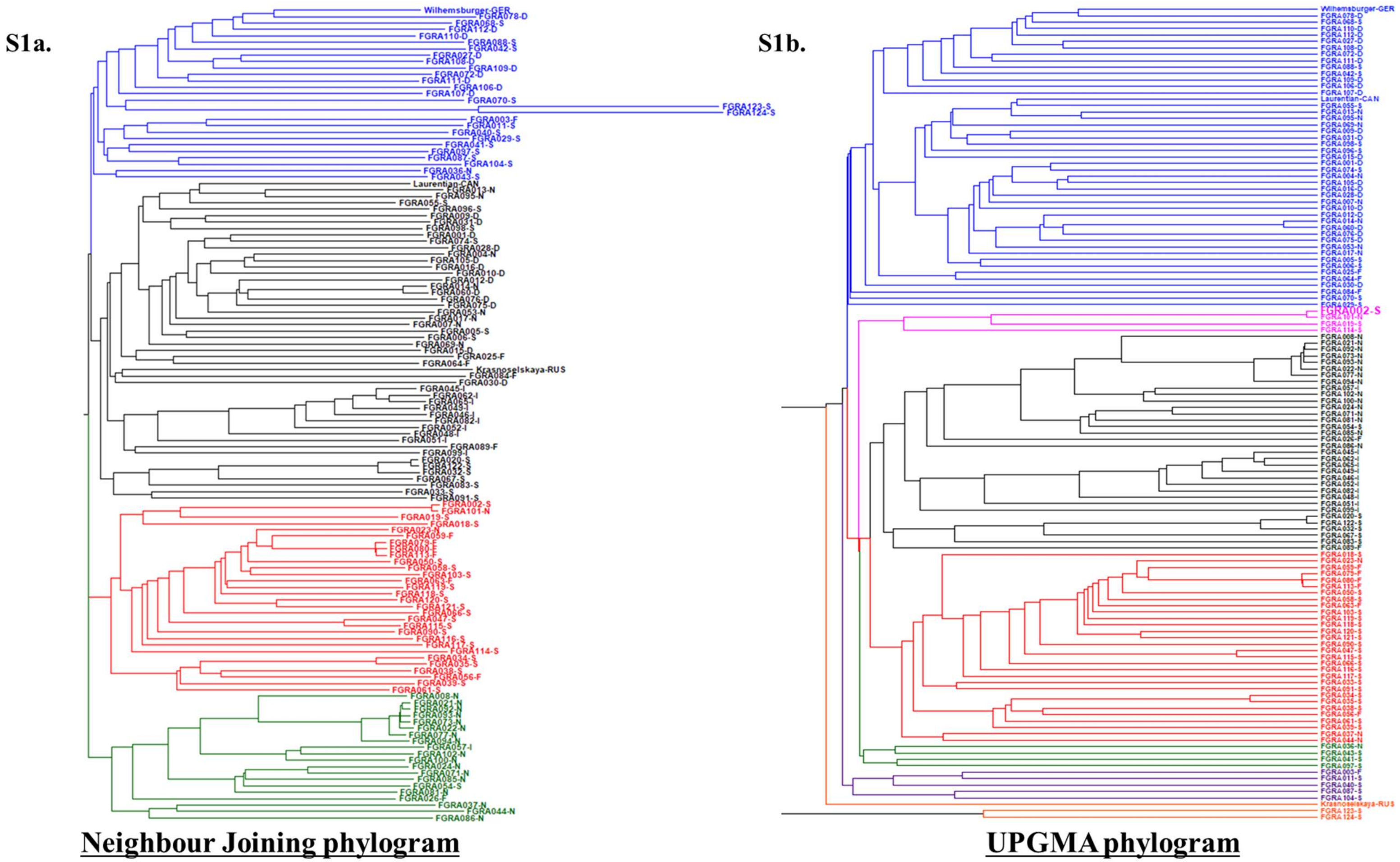

Figure S1. Neighbour joining (NJ) **(A)** and Unweighted pair group method with arithmetic mean (UPGMA) **(B)** phylograms with 6861 SNP markers grouped the 124 rutabaga accessions from Norway, Sweden, Finland, Denmark and Iceland into 4 and 5 subgroups, respectively.

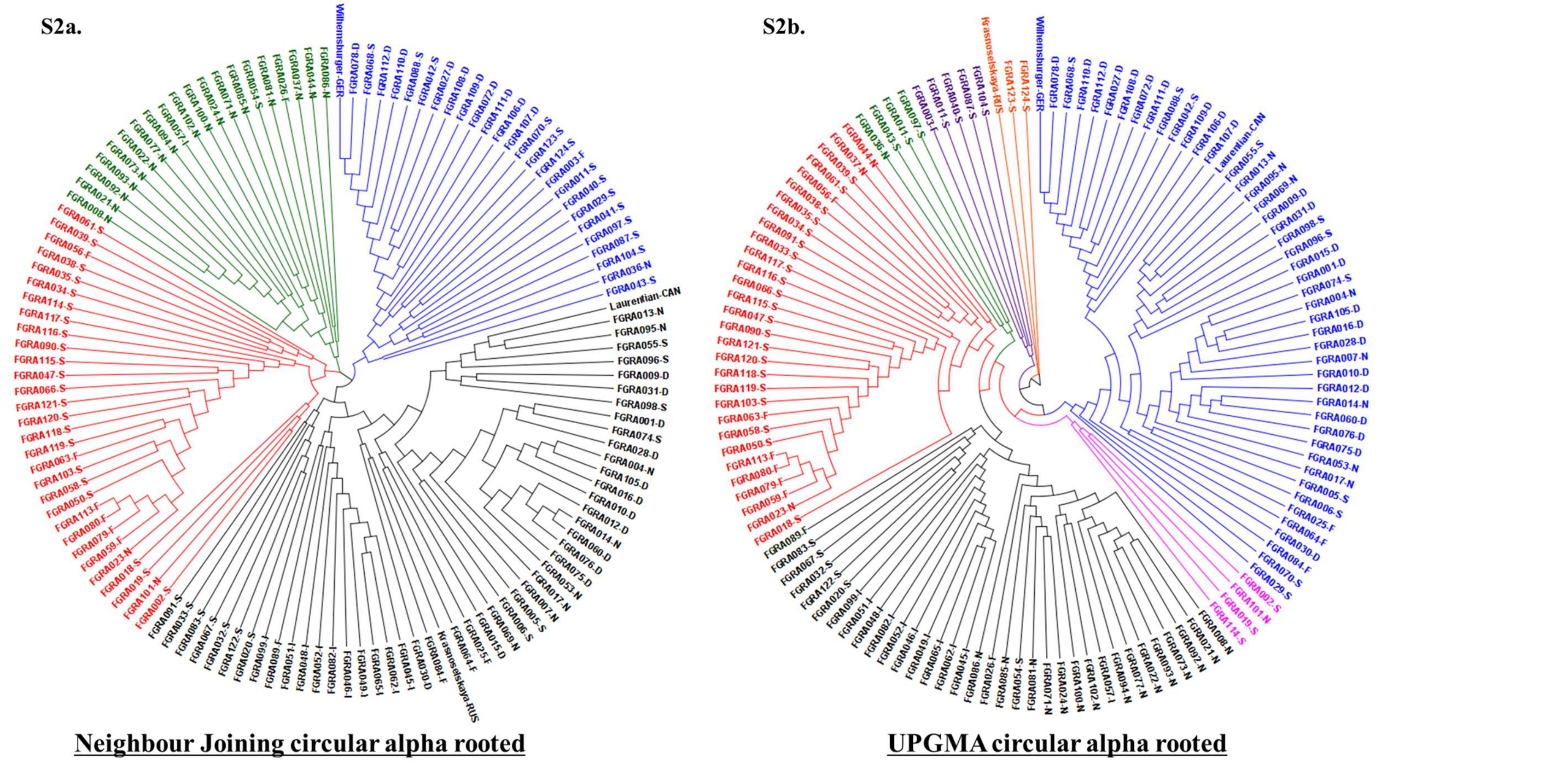

Figure S2. Neighbour joining (NJ) **(A)** and Unweighted pair group method with arithmetic mean (UPGMA) **(B)** circular alpha rooted cladogram with 6861 SNP markers grouped the 124 rutabaga accessions from Norway, Sweden, Finland, Denmark and Iceland into 4 and 5 subgroups, respectively.

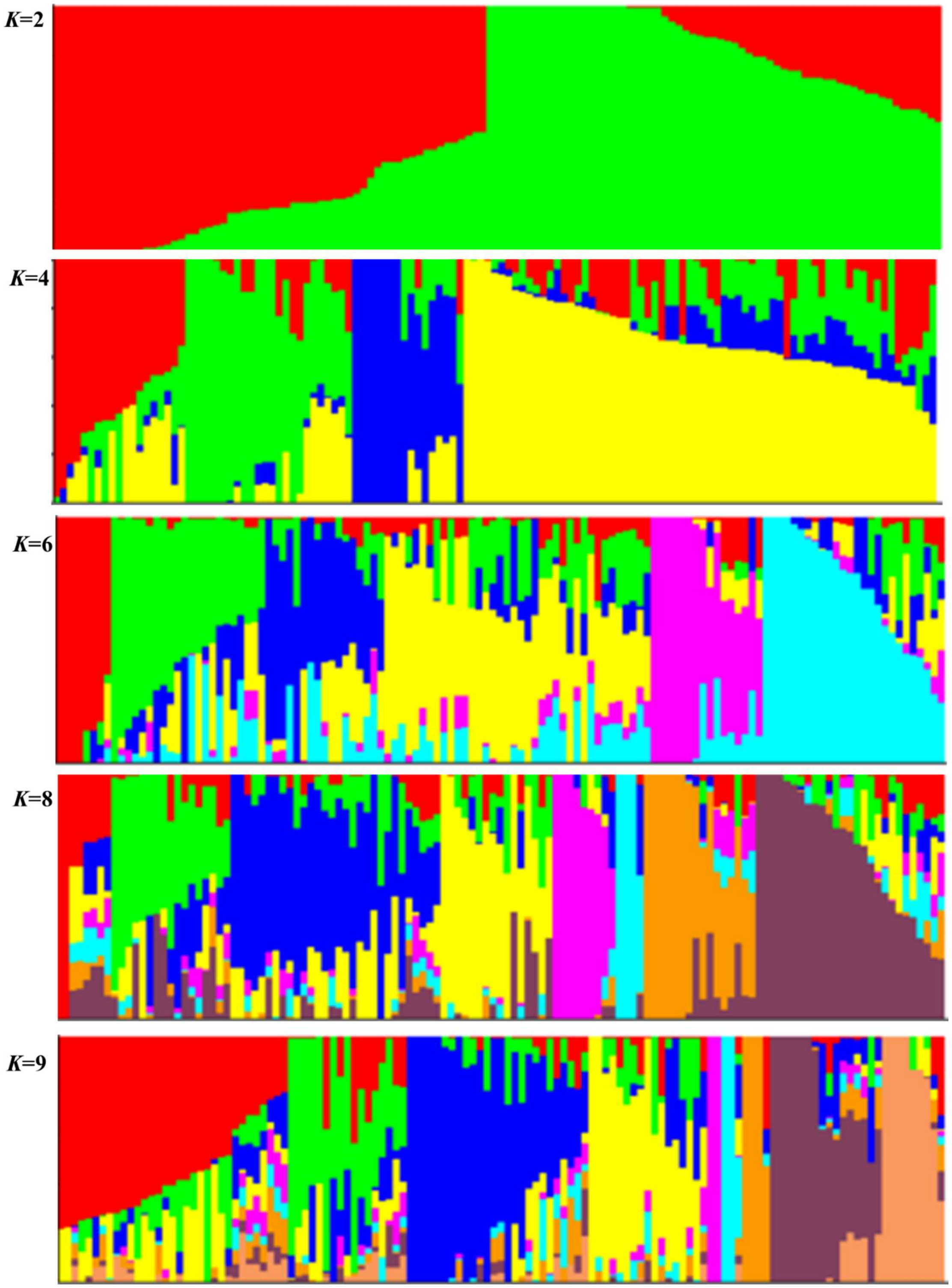

Figure S3. Bayesian cluster analysis of 124 rutabaga accessions from Norway, Sweden, Finland, Denmark and Iceland estimated using the software *STRUCTURE* based on 6861 SNP markers.
